# Current iodine nutrition status and progress toward elimination of iodine deficiency disorders in Jazan, Saudi Arabia

**DOI:** 10.1186/1471-2458-12-1006

**Published:** 2012-11-20

**Authors:** Rashad Mohammed Ali Alsanosy, Abdelrahim Mutwakel Gaffar, Husam Eldin Elsawi Khalafalla, Mohamed Salih Mahfouz, Abdel Naser Shaaban Zaid, Ibrahim Ahmed Bani

**Affiliations:** 1 Family and Community Medicine Department, Faculty of Medicine, Jazan University, PO Box 2531, 45142, Jazan, Saudi Arabia; 2 Medical Research Centre, Jazan University, Jazan, Saudi Arabia; 3 Member of the International Council for Control of Iodine Deficiency Disorders (ICCIDD), Ottawa, ON, Canada; 4 Surgery Department, Faculty of Medicine, Jazan University, Jazan, Saudi Arabia

**Keywords:** Iodine nutrition, Saudi Arabia, Jazan, USI

## Abstract

**Background:**

The term iodine deficiency disorders (IDD) refers to all the effects of iodine deficiency on growth and development in human and animal populations that can be prevented by correction of the iodine deficiency. The objective of this paper was to determine the iodine nutrition status among schoolchildren in the Jazan Region of the Kingdom of Saudi Arabia (KSA), by measuring urinary iodine concentrations and by clinical assessments of goiter rate.

**Methods:**

A school-based cross-sectional survey was conducted in the Jazan region of southwestern KSA from May to November 2010. A total of 311 children, aged 6–13 years, drawn from 12 schools, were selected by a three-stage cluster random sampling method. Data on sociodemographic characteristics were collected using a structured questionnaire. Urine samples were collected and physical examinations were conducted to determine the presence or absence of goiter. Data were analyzed using SPSS version 17.0. Chi square and independent *t*-tests were used for proportions and mean comparisons between groups.

**Results:**

Out of 360 selected children, 311 were examined. There were 131 males (42%) and 180 females (58%). The median urinary iodine concentration (UIC) of the study group was 421 μg/L. The study population proportion with UIC > 300 μg/L was 74% with a higher proportion among males and urban populations. The proportion of children with UIC of 100–300 μg/L was only 21% and was significantly higher among females compared with males (*p* < 0.001). Only about 3% of the children had a median UIC less than 50 μg/L. The prevalence of total goiter rate (TGR) among the sample of schoolchildren in Jazan was 11%, with significant variations between rural and urban populations and by gender.

**Conclusions:**

The present study demonstrates a remarkable achievement in Universal Salt Iodization (USI) and IDD elimination goals in the Jazan area. However, UIC levels reflect excessive iodine intake and may put the population at risk of adverse health consequences like iodine-induced hyperthyroidism and autoimmune thyroid diseases.

## Background

The term iodine deficiency disorders (IDD) refers to all the effects of iodine deficiency on growth and development in human and animal populations that can be prevented by correction of the iodine deficiency [1]. IDD in the most severe form includes cretinism, stillbirth and miscarriage, and increased infant mortality [1]. In developing countries about 38 million newborns every year remain unprotected from the lifelong consequences of brain damage associated with IDD [2]. A recent study estimated that 266 million school-age children and two billion of the general population worldwide have insufficient iodine intake [3].

In the Middle East and North Africa region the situation of IDD control varies considerably between countries. Only the Islamic Republic of Iran and Tunisia have achieved IDD control goals [4]. Iraq, Afghanistan and Pakistan were classified as suffering from severe IDD while Morocco, Sudan and the Kingdom of Saudi Arabia (KSA) were considered as suffering from moderate IDD problems [4,5].

Universal Salt Iodization (USI) is a strategy to ensure sufficient intake of iodine by all individuals was recommended by the WHO and UNICEF Joint Committee on Health Policy in 1994 [6]. Some experts believe that universal salt iodization may be the most successful public health effort of the past two decades [2] and a remarkably cost-effective public health goal [7].

UNICEF estimates that less than 20% of households in the developing world were using iodized salt in the early 1990s, and by 2000, the average had jumped to some 70%. By 2006, around 120 countries were implementing salt iodization programs [2]. In 2007, worldwide 12 countries have optimal iodine status and iodine intake is more than adequate, or even excessive, in 34 countries [3], an increase from 27 in 2003 [8]. In the Middle East and North Africa region, 64% of households consume adequately iodized salt with wide variation between countries [5].

The targets for sustainable IDD elimination include process and impact indicators. The process indicators are; (1) 100% of all salt produced and imported is iodized; (2) 95% of all iodized salt imported and produced is adequately iodized; (3) 90% of households are using adequately iodized salt; and (4) 100% of all districts have reached the goal of USI, i.e. > 90% of households are using adequately iodized salt. The impact indicators are: (1) population median urinary iodine concentration (UIC) is 100–300 μg/L; (2) proportion of samples with UIC levels below 100 μg/L is < 50%; and (3) proportion of samples with UIC levels below 50 μg/L [6].

In the KSA a national cross-sectional epidemiological survey for studying iodine status was conducted among Saudi schoolchildren aged 8–10 years in 1997 [9]. The median national UIC was 180 μg/L and the total goiter rate (TGR) ranged from 8% to 30%. Nationally the proportion of the population with UIC less than 100 was 23%. The southern province of Jazan had the lowest median UIC (110 μg/L) and the highest percentage (45%) of subjects with a UIC < 100 μg/L [9]. The IDD control program using the USI strategy started in 1997, and the Saudi Standards, Metrology and Quality Organization (SASO) recommend that iodine content of salt must be 70–100 ppm in all food salt [10]. Since the national survey in 1997, no follow up survey or monitoring system has taken place [4] to assess the iodine nutrition status in the population neither nationally or in the Jazan region.

The objective of this paper is to determine the iodine nutrition status among schoolchildren in the Jazan Region of the KSA, by measuring the UIC and by clinical assessment of the goiter rate.

## Methods

### Setting

This was a cross-sectional, observational study conducted in the Jazan region during the period from May to November 2010. The Jazan region is one of the 13 regions of the KSA, located on the tropical Red Sea coast in the southwestern KSA. Jazan covers an area of 11,671 km^2^, including some 5,000 villages and towns. Adjacent to the region are 100 islands, including the largest island of Farasan. The Jazan region runs along the Red Sea coast for almost 300 km and is a highly relatively populated region with a total population of 1.5 million.

### Sampling method

A three-stage cluster random sampling procedure was adopted to select target children from 12 schools representing different governorates of the Jazan region. The 12 schools were randomly selected from the list of schools of the target age group in the region and then one class was randomly selected in each of the selected schools. Proportionate to Population Size (PPS) sampling technique was used to determine the required number of children from classes as recommended by the WHO for iodine nutrition surveys. We aimed to obtain a sample of 300 children for this study [6].

### Data collection tools and techniques

Data regarding participants’ sociodemographic characteristics were collected from the children using a structured questionnaire. A casual urine sample of ~10 mL also was collected in a plain tube from each child participating in the survey and stored in a refrigerator (4°C). An aliquot of urine (5–6 mL) was transferred to a tube with a tight cap and was shipped to the International Council for the Control of Iodine Deficiency Disorders (ICCIDD) iodine reference laboratory in the Nutritional Intervention Research Unit (NIRU) of the Medical Research Council (MRC), Cape Town, South Africa, for determination of iodine concentrations. Another aliquot of urine (5–6 mL) was kept as a reference material at the Jazan University Medical Research Centre.

Urine samples were analyzed using a modified microplate method based on manual digestion with ammonium persulfate followed by the colorimetric determination of the Sandel-Kolthoff reaction by using 96-multiwell plates and an absorbance microplate reader at 405 nm. The absolute iodine value is expressed in μg/L [6,11].

### Quality control

Internal quality control (QC) samples were prepared by collecting random urine samples over a 24-hour period, which were either diluted with deionized water or spiked with a known potassium iodate solution to obtain samples at low, median and high iodine concentrations. These QC samples are normally used to determine the inter- and intra-assay precision of the method. During the analysis of study samples the analytical intra-assay precision of the method was determined by calculating the coefficient of variation (% CV) of four analyses of six urine pools ranging from 49.6 to 239.8 μg/L. Inter-assay precision was established by assaying three urine pools (low, medium and high) in 8 to 12 microplate runs. The intra-assay CVs were 5.0%, 2.2%, 0.5%, 1.4%, 0.6% and 6.6% at 49.6, 100.0, 120.7, 129.2, 175.2 and 239.8 μg/L iodine, respectively. The inter-assay CVs were 7.8%, 4.8% and 7.1% at 47.9, 124.6 and 230.4 μg/L iodine, respectively.

### Clinical assessment

Neck examination for goiter was done by trained medical doctors for all children. The ICCIDD uses a simplified goiter classification: grade 0, no palpable or visible goiter; grade 1, palpable but not visible when neck is in normal position; and grade 2, palpable and visible when the neck is in normal position [6].

### Statistical analysis

Results are presented as means ± standard deviation (SD), medians and frequencies as well as percentages. Comparisons between two means were conducted using Student’s *t*-test for continuous variables. The chi-square test was used to compare some selected categorical variables. Before the *t*-test was used, normality assumptions were assessed using the Kolmogorov–Smirnov test and homogeneity of variances were tested using the Levene’s test. All statistical analyses were performed using SPSS, version 17 (SPSS Inc., Chicago, IL, USA). *P*-value less than 0.05 was considered statistically significant.

### Ethical considerations

Ethical approval was obtained from the Research Ethics Committee in the Medical Research Center-Jazan University, and the study was approved by the regional education authorities. Written informed consent was obtained from the parents of participating children.

## Results

Out of 360 selected children 311 were examined and provided salt and urine samples (response rate = 86%). The mean age of the children was 10 years (SD 1.6), and ranged between 6 to 13 years. There were 131 males (42.1%) and 180 females (57.9%). Rural dwellers formed the biggest proportion of children (72.2%); the urban population constituted (24.8%) of the children.

Table 1 presents the number of participants, median, mean and SD of UIC and percentage of participants with UIC > 300 μg/L. The median UIC was 517 μg/L and was significantly higher among male participants (*P*-value < 0.001). However, no significant difference (*P* = 0.230) was observed between the mean UIC for rural and urban children. The proportion of the population with UIC > 300 μg/L was 74% with a higher proportion among males and urban populations.

**Table 1:** The sample size, median, and mean (SD) urinary iodine concentration (UIC) and percentage of subjects with urinary iodine concentration > 300 μg/L

**Item**	**Urinary iodine concentration (μg/L)**				**% with iodine concentration > 300 μg/L**	
	**N**	**Median**	**Mean (SD)**			
**Gender**						
Male	131	484	516 ± 213	*P* < 0.001	88	*P* < 0.001
Female	180	369	399 ± 245		64	
**Residency**						
Rural	234	415	440 ± 230	*P* = 0.230	71	*P* < 0.05
Urban	77	436	475 ± 200		83	
Total	311	420	449 ± 239		74	

Table 2 shows the UIC (μg/L) of school children according to residence and gender in the Jazan region. The proportion of the population with UIC 100–300 μg/L was only 21% and was significantly higher among females than males (*P* < 0.001). Only 3% of the population had a UIC less than 50 μg/L. The proportion of the population with a UIC more than 200 μg/L was 87%.

**Table 2:** Urinary iodine concentration (μg/L), according to residence and gender in Jazan

**UIC range**	**Residence no. (%)**		**Gender no. (%)**		**Total**
	**Rural**	**Urban**	**Male**	**Female**	
	***n*=234**	***n=*77**	***n*=131**	***n*=180**	
0-49	8 (3.4)	-	-	8 (4.4)	8 (2.6)
50-99	7 (3.0)	-	1 (0.8)	6 (3.3)	7 (2.3)
100-199	22 (9.4)	3 (3.9)	5 (3.8)	20 (11.1)	25 (8.0)
200-299	30 (12.8)	10 (13.0)	10 (7.6)	30 (16.7)	40 (12.9)
≥300	167 (71.4)	64 (83.1)	115 (87.8)	116 (64.4)	231(74.3)
*P*-value	0.085		< 0.001		

Table 3 shows the distribution of schoolchildren according to goiter prevalence. The prevalence of TGR among schoolchildren in Jazan was 11% with significant variations between rural and urban populations and gender. The TGR was higher among rural than urban population (*P* = 0.004) and higher in female than male participants (*P* = 0.012).

**Table 3:** Distribution of schoolchildren according to goiter prevalence

**Goiter rate**	**Residence no. (%)**		**Gender no. (%)**		**Total**
	**Rural**	**Urban**	**Male**	**Female**	
Grade 0	202 (86.3)	73 (100)	121 (95.3)	145 (85.6)	275(89.6)
Grade 1	18 (7.7)	-	5 (3.9)	13 (7.2)	10 (5.9)
Grade 2	14 (6.0)	-	1 (0.8)	13 (7.2)	14 (4.6)
Total	234 (100)	73 (100)	127(100)	180 (100)	307(100)
*P*-value	0.004		0.012		

## Discussion

The three most important indicators used in IDD surveillance, as well as in initial problem assessment, are goiter prevalence, urinary iodine excretion in a population, and thyroid function tests [6]. The WHO advises researchers to combine at least two indicators, one morphological and one laboratory test. A 1997 survey found that 23% of the national population and 45% of the Jazan region population had UIC below 100 μg/L, although their median urinary iodine concentration was within the normal range. Additionally, goiter prevalence ranged from 25% to 30% in costal and mountain areas in the southern region. The authors recommend that the country needs to implement an IDD control program [9]. In this study the researchers used both urinary iodine excretion and goiter prevalence to assess the iodine nutrition status.

### Urinary iodine

In our study the median UIC among the studied population in the Jazan area was 421 μg/L compared with 110 μg/L in the 1997 survey in Jazan, and 180 μg/L at the national level [9]. This median UIC reflects excessive iodine intake in the population based on WHO cut-off points [6]. The median UIC is significantly higher among male participants (484 μg/L) than female participants (369 μg/L) (*P*-value < 0.001). The median UIC in Jazan, KSA, is the highest in the Gulf and Arab region and similar to that reported in Ecuador (420 μg/L), Uganda (464 μg/L) and the Democratic Republic of the Congo (495 μg/L) [5].

Based on the UIC cut-off points recommended by the WHO regarding iodine nutrition status, only 8% of the population has adequate iodine intake (UIC 100–199 μg/L), 13% above requirements (UIC 200–299 μg/L), and 74% with excessive iodine intake [6]. Only 5% of children have UIC less than 100 μg/L compared with 45% in 1997 [9].

The population of Jazan was subjected to excessive iodine intake with a risk of adverse health consequences like iodine-induced hyperthyroidism and autoimmune thyroid diseases [6,12-14]. A similar phenomenon was observed in 34 countries after the implementation of USI, which emphasized the importance of establishing and strengthening the monitoring system to guide USI to provide optimal iodine nutrition and protect against excessive intake [6,15].

### Goiter rate

Goiter prevalence in the studied region ranged from 0% in urban areas to 14% in rural areas. The prevalence of goiter was significantly higher (*P*-value = 0.012) among female participants (14%) than male participants (11%). Al-Nuaim et al. in 1997 reported a goiter prevalence rate of 4% in Gizan or Jazan and 22% in the Aseer area [9]. Similar prevalence in the Aseer region (24%) was reported by Abu-Eshy et al. in 2001 [16]. Goiter prevalence in Jazan is much less than what is reported by many countries in the Eastern Mediterranean region. In Yemen the TGR was 16.8% [5,17]. In the Gulf region the TGR in 2010 was 40.4% in the United Arab Emirates, 10% in Oman and 1.7% in Bahrain [5]. On the other hand in other Arab countries the TGR is 65% in Algeria, 36.3% in Tunisia, 33.5% in Jordan, 30.9% in Mauritania, 25.7% in Lebanon, and 21.4% in Egypt [5]. In Iran, the TGR was reduced from 68.0% in 1989 to only 5.7% in 2007 through implementation of a comprehensive IDD control program [18].

The salt iodization standard in the KSA as issued by SASO recommends that the amount of iodine to be between 70–100 ppm in the form of potassium iodate. This recommendation is the highest in the Gulf region [10] and higher than that recommended by the WHO which is 15–40 ppm [6]. Since the start of the salt iodization program in 1997, now more than 99% of the study population in Jazan uses iodized salt. Three hundred and eleven random salt samples were tested to determine the iodine content; 44.1% had iodine concentrations higher than 40 ppm.

Al-Attas quantified the iodine content of foods in the KSA and concluded that food consumed by Saudis appears to have an adequate iodine concentration [19]. The high level of UIC may be explained, in light of the previous study finding by Al-Attas, by the high level of iodine concentrations in salt, increased consumption of dairy products, and ready-prepared salty meals that may have substantially changed the amount of salt and iodine consumed by the school population.

There is a need to reconsider the level of salt iodization in the KSA following the latest WHO recommendation in light of local dietary patterns. It will be important to provide optimal iodine nutrition and to protect the population from the adverse health consequences of excessive iodine intake.

## Conclusion

The present study demonstrates a remarkable progress in achieving USI and IDD elimination goals in the Jazan area of the KSA. However, the UIC reflects excessive iodine intake and may put the population at risk of adverse health consequences like iodine-induced hyperthyroidism and autoimmune thyroid diseases. The levels of iodine in the salt were substantially higher than the WHO recommendation and revision of salt specifications is highly recommended. Establishing surveillance and monitoring systems will protect the population and help in guiding the implementation of USI in the country.

### Study limitations

Logistical issues of the study including send samples to South Africa for analysis. For this reason the sample size is small to detect the differences between clusters, although it is quite reasonable for the study. Now that the capacity of the Medical Research Center is developed, further studies could be done and samples analysis will be conducted locally. The schools in the country do not provide meals for their students. Also, no school nutrition program is implemented in the country and the food industry is required to use iodized salt for all processed food. Thus, the results reflect the iodine nutrition in the community.

## Competing interest

The authors have no conflicts of interest to declare.

## Authors’ contribution

RMA, HEE, ASZ and IAB prepared the project proposal; RMA, AMG and MSM designed this research paper; MSM and AMG performed data analysis; AMG and RMA wrote the manuscript. HEE, IAB, and ASZ provided significant input on the manuscript. All authors read and approved the final manuscript.

## Sources of funding

This work was funded by the Medical Research Center, Jazan University, KSA, under grant project No. 1000.

## Pre-publication history

The pre-publication history for this paper can be accessed here:

http://www.biomedcentral.com/1471-2458/12/1006/prepub
